# Supplementary material for: Analysis of chi angle distributions in free amino acids via multiplet fitting of proton scalar couplings
Source: Magn Reson (Gott). 2024 Aug 19;5(2):103–20. doi: 10.5194/mr-5-103-2024 (PMC11570886; doi:10.5194/mr-5-103-2024)
Supplement: The supplement related to this article is available online at: https://doi.org/10.5194/mr-5-103-2024-supplement. [file mr-5-103-2024-supplement.zip › Syed2024/data/fit1d_gissmo.tar.gz!/fit1d/glu/500/bmse000037_simulation_1_nmredata.zip!/bmse000037_simulation_1_nmredata/1H/pdata/1/index.html_C=S_O=A]

Index of /ftp/pub/bmrb/metabolomics/standards/L\_glutamic\_acid/nmr/bmse000037/1H/pdata/1


# Index of /ftp/pub/bmrb/metabolomics/standards/L\_glutamic\_acid/nmr/bmse000037/1H/pdata/1

|  | Name | Last modified | Size | Description |
| --- | --- | --- | --- | --- |
| --- | | | | |
|  | Parent Directory |  | - |  |
|  | hwcal.out | 03-Oct-2011 11:05 | 12 |  |
|  | title | 03-Oct-2011 11:05 | 59 |  |
|  | intrng | 03-Oct-2011 11:05 | 65 |  |
|  | peakrng | 03-Oct-2011 11:05 | 330 |  |
|  | outd | 03-Oct-2011 11:05 | 495 |  |
|  | auditp.txt | 03-Oct-2011 11:05 | 1.7K |  |
|  | procs | 03-Oct-2011 11:05 | 1.7K |  |
|  | proc | 03-Oct-2011 11:05 | 1.8K |  |
|  | peaklist.xml | 03-Oct-2011 11:05 | 2.2K |  |
|  | peaks | 03-Oct-2011 11:05 | 2.5K |  |
|  | 1i | 03-Oct-2011 11:05 | 128K |  |
|  | 1r | 03-Oct-2011 11:05 | 128K |  |
| --- | | | | |

Apache/2.2.3 (CentOS) Server at www.bmrb.wisc.edu Port 80
